# Efficient Phytoremediation of Methyl Red and Methylene Blue Dyes from Aqueous Solutions by *Juncus effusus*

**DOI:** 10.1021/acsomega.4c07468

**Published:** 2025-01-10

**Authors:** Oya Aydın Urucu, Benedetta Garosi, Rabi A. Musah

**Affiliations:** † Department of Chemistry, University at Albany − State University of New York (SUNY), 1400 Washington Avenue, Albany, New York 12222, United States; ‡ Department of Chemistry, Marmara University Faculty of Sciences, Istanbul 34722, Turkey

## Abstract

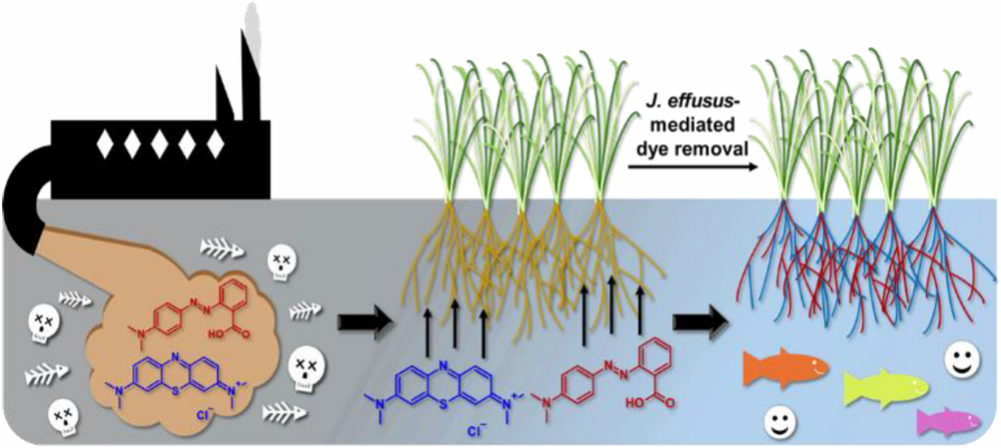

The contamination of water with dyes stemming from the discharge of industrial waste poses significant environmental risks and health concerns. In this study, the phytoremediation potential of the wetland plant *Juncus effusus* was investigated (as a function of plant biomass, pH, contact time, and initial dye concentration) for the removal of methylene blue and methyl red dyes from wastewater. The experimental adsorption capacities under the optimum conditions were found to be 1.421 and 1.038 mg g^–1^ plant wet weight for methylene blue and methyl red, respectively. Pseudo-first-order and pseudo-second-order models were employed to determine the kinetics of the phytoremediation adsorption process. The pseudo-second-order model was found to be the most suitable for both methylene blue and methyl red. The results were found to conform to the Freundlich equilibrium isotherm, with a correlation of *R*^2^ ≥ 0.99 for removal of both dyes. Confirmation of dye uptake by the plant was determined by using Fourier transform infrared spectroscopy (FTIR). Additionally, direct analysis in real time–high-resolution mass spectrometry (DART-HRMS) analysis of plant roots is reported here for the first time as a means to investigate dye degradation by plant roots.

## 1 Introduction

Among the most widespread water contaminants are persistent organic dyes,^1−3^ which are widely used as coloring agents in the textile, food, printing, and cosmetic industries. Chronic exposure to these substances is hazardous for humans, terrestrial animals, and aquatic flora and fauna. Dyes are known to be carcinogenic and cause susceptibility to infection and dermatitis in humans. In aquatic environments, they also inhibit the photosynthetic activities of plants and reduce biodiversity by blocking light entry.^4−8^ Consequently, effective approaches for the elimination of dye compounds from water are of critical importance.^9−11^

Several physical and chemical methods have been employed to treat effluents contaminated with dyes. These include membrane filtration,^12^ sedimentation,^13^ adsorption,^14^ sonochemistry,^15^ ion exchange,^16^ and coagulation^17^ among others. Yet, due to their high cost and/or the generation of additional pollutants that can accompany the application of some of these approaches, widespread adoption of these methods has been slow.^5^ For this reason, considerable attention is being directed to the development of environmentally benign approaches, such as phytoremediation, for the removal of dye contaminants from water bodies. Phytoremediation utilizes plants to remediate polluted environments such as soil and water via the uptake of pollutants through the roots. Phytoremediation and phytomanagement, when integrated with sustainable site management, not only offer environmental benefits, but also provide economic and social well-being. Furthermore, it has been linked to other ecosystem services, such as nutrient recycling, efficiency, carbon sequestration, water movement, and water purification. Beyond merely reducing pollution, phytoremediation aims to restore the quality and functionality of resources.^18,20,21^ Besides being an aesthetically pleasing treatment approach, it can be accomplished at significantly reduced cost, and depending on the plant species utilized, it can even generate bioenergy.^18^ Mechanisms by which plants successfully remove compounds from water and soil include phytoextraction and rhizofiltration, dissipation (phytovolatilization), degradation (rhizodegradation and phytodegradation), and immobilization (hydraulic control and phytostabilization) to immobilize, degrade, or remove pollutants. Different plants utilize one or a combination of these mechanisms.^18,19^ The choice of which to use depends on the type of pollutant and the matrix (i.e., soil or water). Other considerations include the suitability of the plant to the local climate, its capacity to yield high biomass, the ability to extract contaminants, the unpalatability of the plant to herbivores, and ease of cultivation.^18^

In recent years, an increasing number of plant species have been proposed for the removal of organic dye contaminants from water sources. For instance, Sharma et al. have used *Eichhornia crassipes* (Mart.) Solms (water hyacinth) for removal of a range of different cationic and anionic dyes.^22^ Jothilingam et al. have investigated the ability of the aquatic plant *Salvinia molesta* to remove crystal violet dye;^23^ Kaur et al. utilized the ornamental plant *Trachyspermum ammi* L. to remove methylene blue and Congo red dyes;^24^ Mahajan and Kaushal examined the submergent freshwater macroalgae *Chara vulgaris* L. for the removal of methyl red;^25^ and Krishnaswamy et al. used the aquatic macrophyte *Pistia stratiotes* for the removal of mixed azo dyes.^26^ Moreover, Chandanshive et al. studied the ability of garden ornamental plants to remove dyes from textile wastewater, while Ahila et al. investigated dye removal utilizing freshwater macrophytes.^27,28^ While these approaches have shown promise, their broad adoption has been hampered by the limited range of habitats in which the indicated plants can be cultivated. Therefore, there remains a strong need for the development of a phytoremediation system that can be cultivated in a diverse range of climates and geographic conditions and in a variety of matrices (e.g., water, land, and wetlands). Other desirable characteristics include being nonhazardous, noninvasive, and of low cost.^29−31^

One species that fits this profile is *Juncus effusus*. A member of the Juncaceae family (commonly known as “rush”), it is an evergreen ornamental grass that has a broad geographic distribution, being native in Europe, Asia, Africa, Madagascar, North America, and South America, and naturalized in Australia, New Zealand, South Africa, and various oceanic islands.^32^*J. effusus* is native to a large portion of the world’s temperate climates. It prefers full sun but can tolerate some shade.^33^ Furthermore, it has horticultural appeal, is of manageable size, and readily withstands challenging environmental and weather types.^34^ Therefore, the ability of *J. effusus* to adapt to a range of climatic conditions, its high tolerance for poor soils, as well as water-logged areas, and the fact that it is a perennial are factors that may enhance its potential suitability as a phytoremediator. Because of the three-dimensional network of its biomass, numerous natural sorbents have been developed from its tissue,^34−41^ but remarkably, the live plant itself has not previously been reported for dye removal from polluted waters by the phytoremediation process. Described here are the results of an investigation of the effectiveness of *J. effusus* for removing methyl red (MR) and methyl blue (MB) from contaminated water. The adsorption process for the uptake of MB and MR was examined as a function of pH, contact time, and initial dye concentration. Adsorption isotherm and kinetics studies, as well as infrared (IR) and direct analysis in real time–high-resolution mass spectrometric (DART-HRMS) analyses, were also conducted to characterize the mechanism of dye removal.

## 2 Materials and Methods

### 2.1 Materials

Methylene blue and methyl red were purchased from Sigma-Aldrich (St. Louis, MO, USA) and Fisher Scientific (Pittsburgh, PA, USA), respectively. To prepare dye stock solutions with a concentration of 1000 mg L^–1^, 0.5 g of dye was first dissolved in 25 mL of ethanol (Pharmco-Aaper, Brookfield, CT, USA) and then diluted to 500 mL with distilled water. The nutrient medium used for the cultivation of the plants was prepared using Hoagland solution (Plant Media, Dublin, OH, USA). *J. effusus* was purchased from a perennial farm marketplace on Amazon.com.

### 2.2 Adsorption of Organic Dye

Upon receipt, *J. effusus* plants were cleaned with tap water to remove soil, placed in Hoagland nutrient solution for 24 h, and then washed again with pure water prior to being used in experiments. Experiments were conducted in batch mode to investigate the effects of plant biomass (assessed in terms of plant weight), pH, contact time, and initial dye concentration on the phytoremediation potential of *J. effusus* with both MB and MR dyes. To examine the effects of increasing plant biomass and to assess the impacts of initial dye concentration, contact time, and pH, multiple plants with the same approximate weight, and a height of ∼20 cm were used. Plants were exposed to dye-contaminated water by immersing the roots into 50 mL of dye solution at concentrations of 10, 20, 30, 40, and 50 mg L^–1^ and pH values varying from 2 to 9 contained within 125 mL Erlenmeyer flasks. Dilute HCl and KOH solutions were employed for making pH adjustments. The concentration of the dye solutions used to determine the impact of exposure/contact time and assessment of the pH of optimal adsorption was 20 mg L^–1^. At specified time intervals of up to 80 h, aliquots were removed to make absorbance measurements using a UV−visible spectrophotometer (PerkinElmer Lambda-35, Waltham, MA, USA). All studies were conducted in a greenhouse under natural light conditions at the University at Albany (Wadsworth Control Systems) maintained at 23 ± 1 °C during the period from March to May 2024. Calibration curves for the determination of dye concentrations in water were created using the maximum absorbance of 664 and 435 nm for MB and MR, respectively. All experiments for kinetic analyses were performed in triplicate (*N* = 3), and calculations were based on the average values obtained from the three replicates.

Equation 1 was used to calculate % Removal (i.e., % Decolorization), and eq 2 was used to calculate adsorption capacity (*q*_e_) of MB and MR by *J. effusus*.^42,43^
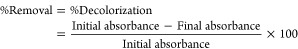
1
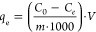
2where *C*_*0*_ and *C*_e_ refer to the initial and post adsorption dye concentration (mg L^−1^), respectively, *V* is the volume of dye solution (mL), *q*_*e*_ is the adsorption capacity of the plant (mg g^–1^), and *m* is the mass of plant material (g).^44^

### 2.3 Determination of Differential Dye Uptake by *J. effusus*

To determine whether there was differential uptake of dye by *J. effusus* as a function of chemical structure, dye adsorption was monitored by exposing live *J. effusus* plants to a 50 mL solution of simulated dye-contaminated water containing a mixture of MB and MR (10 mg L^–1^ each) for 80 h at pH 7.0. The concentration of each dye was adjusted to 10 mg L^–1^, and the pH was set to 7.0. A plant mass of 1.5 g was used in these experiments.

### 2.4 Attenuated Total Reflectance–Fourier Transform Infrared Spectroscopy (ATR-FTIR) Analysis of *J. effusus*

Attenuated total reflectance−Fourier transform infrared spectroscopy (ATR-FTIR) (PerkinElmer Spectrum 100, Waltham, MA, USA) was used to assess interactions between functional groups within molecules contained in *J. effusus* roots and the MB and MR dyes. For these analyses, *J. effusus* roots before (control) and after adsorption were dried in an oven at 50 °C for 24 h. Approximately 20 mg of the root sample was obtained, and this was ground into a fine powder. The powders were then analyzed by ATR-FTIR in the scan range of 650−4000 cm^–1^ at a rate of 10 scans per sample.

### 2.5 DART Mass Spectral Data Acquisition and Analysis

DART-HRMS analysis was conducted on plant roots to investigate the uptake by *J. effusus* of MB and MR dyes. The roots of *J. effusus* (both pre- and postdye adsorption) were collected before and after the phytoremediation process. They were then dried in an oven at 50 °C for 24 h, ground into a fine powder, and subsequently analyzed by DART-HRMS using a capillary tube sampling technique. The closed end of a glass melting point capillary tube was inserted into the powdered sample, and the coated surface of the tube was then exposed to the DART gas stream for about 5 s, resulting in a full mass spectrum which revealed the chemical profile of the roots. This procedure was performed three times for each sample, and the results from these three replicates were averaged to generate a mass spectrum representative of the given sample.

Mass spectra were acquired using a DART-SVP ion source (IonSense, Saugus, MA, USA) coupled to a JEOL AccuTOF high-resolution time-of-flight (TOF) mass spectrometer (Peabody, MA, USA). All analyses were conducted in positive-ion mode with a DART ion source grid voltage of 250 V. Ultrahigh-purity helium was used as the DART gas at a temperature set to 350 °C and a flow rate of 2 L/min. The following parameters were applied: ring lens voltage at 5 V, orifice 1 voltage at 20 V, orifice 2 voltage at 5 V, peak voltage at 600 V, and detector voltage at 2000 V. Data were collected over a mass range of *m*/*z* 60−1000, with PEG 600 used as the mass calibrant for all acquisitions. TSSPro 3.0 software (Shrader Software Solutions, Grosse Pointe, MI, USA) facilitated calibration, spectral averaging, background subtraction, and peak centroiding of the mass spectral data. A millimass unit (mmu) tolerance within ±5 mmu was used to determine the presence of peaks consistent with the masses of the analytes of interest. The Mass Mountaineer software suite (RBC Software, Portsmouth, NH, USA) was used for processing of all mass spectra.

## 3 Results and Discussion

### 3.1 Effect of pH

**Figure 1:**
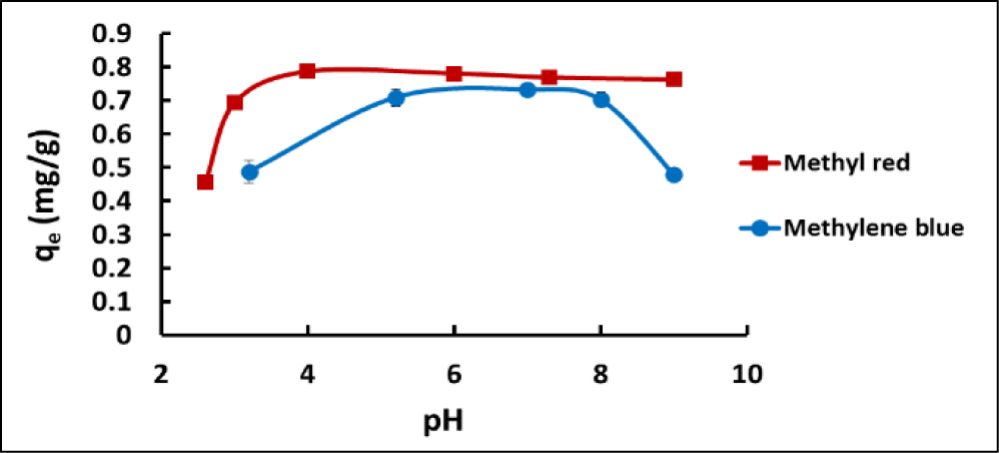
Effect of pH on phytoremediation potential of *J. effusus* for MB and MR using plants of ∼1.0−1.5 g wet weight, with submersion of plant roots in 50 mL dye solutions at a concentration of 20 mg L^–1^.

The influence of pH on the adsorption of MB and MR by *J. effusus* was studied using individual plants (each weighing approximately 1−1.5 g) that were exposed to 50 mL solutions of dye (20 mg L^–1^ concentration) at 5 pH’s between 2 and 9. Representative results are presented in Figure 1, which shows plots of absorption capacity (*q*_e_, see eq 2) as a function of pH for MB (blue) and MR (red). For MB, maximum absorption was observed across the pH range of 5−8, while for MR, maximum absorption occurred between pH 4 and 9. Soil pH is known to significantly impact plant growth for numerous plant species, and many plants achieve optimal growth within a pH range of 6−7.5.^45^Figure 1 reveals that the optimal pH range for *J. effusus* extends between 5 and 8. This aligns with the results of previous studies, which showed that *J. effusus* exhibits a relatively broad tolerance of different soil pH levels.^46,47^ Based on these observations, subsequent *J. effusus* dye removal experiments were conducted at the midpoint pH of 7.0.

### 3.2 Impact of *J. effusus* Biomass on Dye Uptake

**Figure 2:**
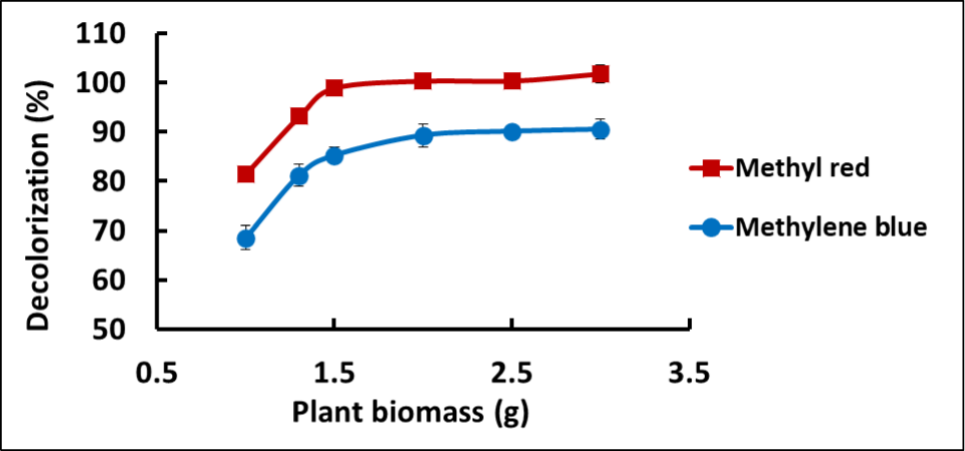
Impact of plant biomass on MB and MR removal from water using plants of ∼1.5 g wet weight. Roots were suspended in 50 mL dye solutions at a concentration of 20 mg L^–1^ and a pH of 7.

To determine the impact of plant biomass on dye uptake, the extent of dye adsorption was quantitatively assessed over a period of 80 h by UV−vis determination of the concentration of dye remaining in solution as a function of plant biomass. Representative results are presented in Figure 2, where those for MB are presented in blue, and those for MR are shown in red. In Figure 2, the *y*-axis indicates the % decolorization, as defined by eq 1, and it reflects the reduction in the absorbance of the dye in the aqueous solution (i.e., decreasing dye concentration in the water) as the biomass increases. For example, for a single plant with a mass of ∼1.0 g wet weight when first introduced to the dye solution, 68.6% of the initial MB dye concentration was taken up by the roots. Analogously, the MR results show that 81.5% of the initial MR dye concentration was taken up over the 80-h period when multiple plants with a total biomass of ∼3.0 g wet weight were exposed to the water. Figure 2 also reveals that the maximum level of uptake of the dyes was ∼85% for MB and ∼99% for MR. This corresponds to concentrations of ∼15% and ∼1% remaining in the aqueous solutions following *J. effusus* exposure. Dye removal by *J. effusus* increased with increasing biomass for up to 1.5 g and then remained unchanged as indicated by the plateau observed in both cases. Thus, in subsequent studies, *J. effusus* plants equivalent to a total mass of ∼1.5 g and approximately 20 cm in height were used.

### 3.3 Impact of Initial Dye Concentration and Plant Exposure Time on *J. effusus*-Mediated Dye Uptake

**Figure 3:**
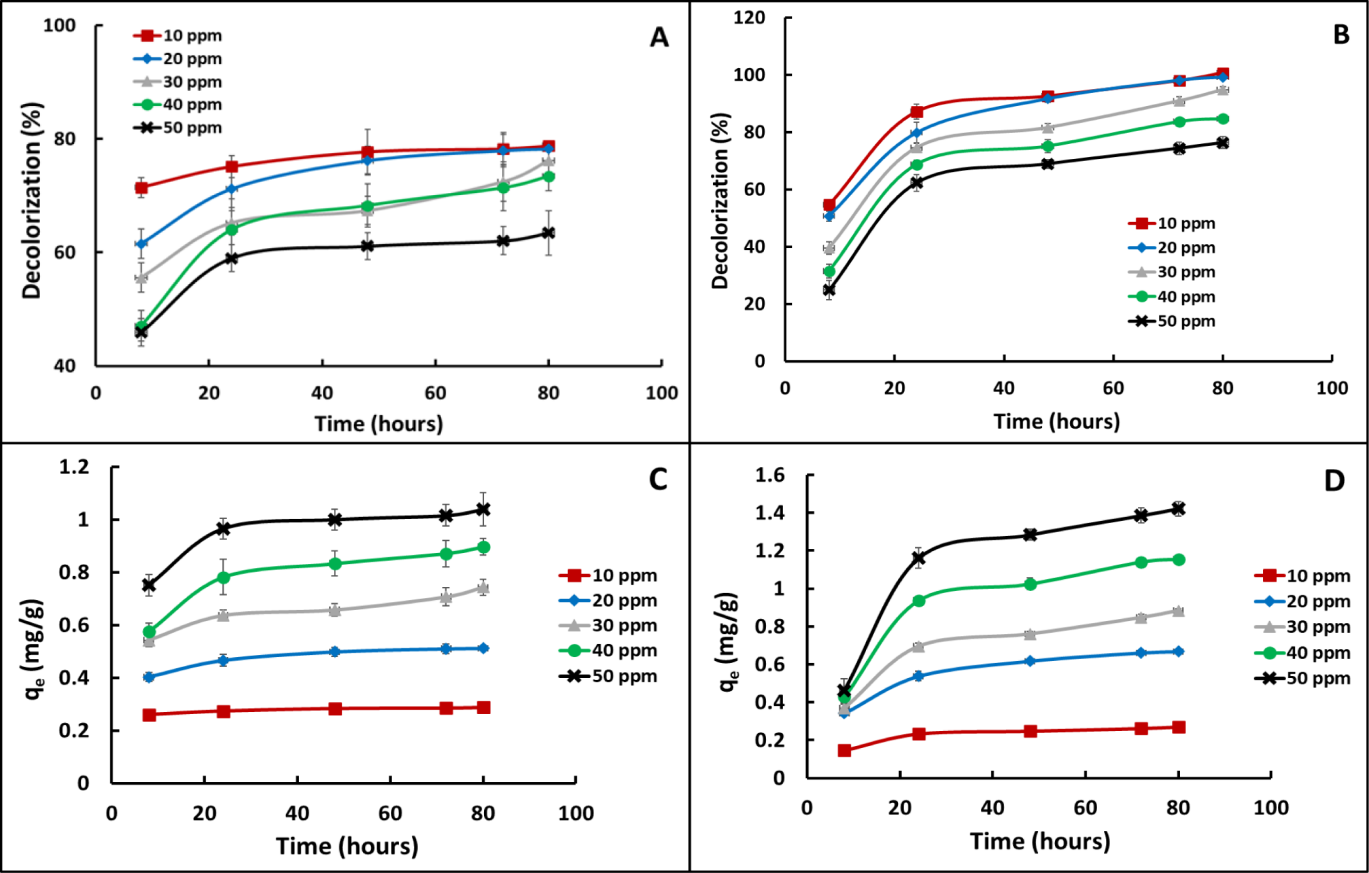
Impact of plant exposure time and dye concentration on uptake by *J. effusus* roots of MB and MR dyes over an 80-h period at pH 7, using plants having a wet weight of ∼1.5 g. A: assessment of dye uptake as measured by percent water decolorization as a function of variations in initial MB dye concentration; B: assessment of dye uptake as measured by percent water decolorization as a function of variations in initial MR dye concentration; C: uptake of MB expressed in terms of adsorption capacity (*q*_e_) as a function of time; and D: uptake of MR expressed in terms of adsorption capacity (*q*_e_) as a function of time.

To assess the impact of both exposure time and dye concentration on the phytoremediation potential of *J. effusus* for MB and MR dyes, the level of uptake of dye as a function of time was monitored through assessment of water decolorization, using the optimized conditions developed previously (i.e., pH 7.0; 1.5 g of wet weight live plants/50 mL of dye solution). The results are presented in Figure 3. Figure 3A shows the level of uptake of MB by *J. effusus* over an 80-h period as a function of different starting concentrations of the dye. The concentrations surveyed were 10, 20, 30, 40, and 50 ppm, indicated with red squares, blue diamonds, gray triangles, green circles, and black x’s, respectively. For example, Figure 3A shows that when the starting MB concentration was 10 ppm (indicated with red squares), the percent decolorization was 71.4% until 8 h, and it increased to 78.8% by 80 h. On the other hand, when the starting concentration of MB was 50 ppm (denoted by black x’s in Figure 3A), the percent decolorization at 24 h was 59%, and dye uptake plateaued by 30 h, after which there was little further uptake through the 80-h period. By 80 h, the MB dye concentration had dropped from 50 to 18.3 ppm. A similar trend was observed for the other concentrations that were surveyed (i.e., 20, 30, and 40 ppm). Figure 3B shows the analogous results for MR. Similar to the case for MB, uptake was most dramatic between hours 0 and 48, after which it reached a plateau, where there was little additional uptake. Figure 3A, B reveals that the maximum percent decolorization for MB and MR (at 10 ppm) was ∼79% and ∼100%, respectively. Another observed trend was that higher starting concentrations of both dyes result in a reduction in the *J. effusus*-promoted decolorization. For example, at 10 ppm of MB, *J. effusus* removed 78.8% of dye after 80 h, while 63.4% was removed at 80 h when the starting concentration of MB was 50 ppm. Nevertheless, the results also show that as the initial dye concentration increases, the amount of dye taken up by the plants also increases. This is revealed in Figure 3C,D which illustrates the uptake of MB and MR dyes expressed in terms of adsorption capacity (*q*_e_) as a function of time. For example, the amount of dye removed from the water sample containing 10 ppm MB (Figure 3C) was 0.287 mg g^–1^, while it increased to 1.038 mg g^–1^ when the MB concentration was 50 ppm. Similar results were observed for the removal of MR (Panel D). For instance, the amount of dye removed from the water sample containing 10 ppm MR was 0.269 mg g^–1^, but it increased to 1.421 mg g^–1^ when the MR concentration was 50 ppm.

### 3.4 Kinetic Studies

One means by which the mechanism of uptake of small molecules by plants has been investigated is through the determination of the kinetic models to which the adsorption behavior conforms (i.e., pseudo-first-order or pseudo-second-order).^3^ Accordingly, to determine the mechanism of adsorption of MB and MR dyes from water by *J. effusus*, both pseudo-first-order and pseudo-second-order kinetics models were assessed. The kinetic constants and parameters corresponding to the linearized equations of these two models are presented in Tables 1 and 2. For assessment of pseudo-first-order kinetics, the data were evaluated by plotting log(*q*_e_– *q*_t_) against time (*t*) to assess linearity, and for pseudo-second-order kinetics, *t*/*q*_t_ was plotted against *t* to assess linearity. The alignment of the kinetic models with the experimental data was assessed based on the regression value (*R*^2^). The results for MB are displayed in Figure S1 (panel A: pseudo-first-order; panel B: pseudo-second-order), and for MR they are shown in Figure S2 (panel A: pseudo-first-order; panel B: pseudo-second-order). From the pseudo-first-order plots, the values of *k*_1_ and *q*_e(theor)_ (i.e., theoretical adsorption capacity in mg g^–1^) were determined from the slope and intercept of the line obtained by plotting log(*q*_e_ – *q*_t_) versus *t*. The *R*^2^ values of these plots ranged from 0.894 to 0.987 for MB and from 0.941 to 0.977 for MR, and the theoretical adsorption capacity values *q*_e(theor)_ were consistently smaller than the experimental values *q*_e(exp)_ as revealed in Tables 1 and 2. This suggests that the pseudo-first-order kinetic model is not as suitable in describing the mechanism of phytoremediation of MB and MR dyes by *J. effusus*. For the pseudo-second-order plots, the intercepts and slopes were used to determine *k*_2_ and *q*_e(theor)_ values for the represented concentrations. The results in Tables 1 and 2 show that the pseudo-second-order kinetic model is most suitable in describing the phytoremediation of MB and MR dyes due to the excellent *R*^2^ values (0.99) and the alignment of the theoretical *q*_e(theor)_ values from the plots with the experimentally observed *q*_e(exp)_ values (i.e., experimental adsorption capacity in mg/g). The conformance of the data to this model indicates that the dye’s dissolved ions are adsorbed through the chemical process of cation exchange or chemical bonding.^21,49^ The rate constant (*k*_2_) value, as shown in Tables 1 and 2, although slightly fluctuating, decreased significantly for both dyes, especially after the initial dye concentration of 20 ppm. This situation may be attributed to the increased competition between dye molecules for the adsorption sites on the surface of the *J. effusus* roots at higher concentrations. Additionally, electrostatic interactions tend to decrease with rising initial concentrations, leading to a reduced affinity of the dye for the plant surface. Therefore, it appears that the chemisorption mechanism of *J. effusus* reduces the phytoremediation efficiency of MB and MR dyes.^25,50^

**Table 1:** Kinetic Parameters for MB Removal by *J. effusus*

			**Initial Dye Concentrations** (mg/L)				
Kinetic Model	Equations (MB)	Kinetic Parameter	10	20	30	40	50
**Pseudo-first-order**		***q*_e(exp)_(mg/g)**	0.287	0.511	0.743	0.896	1.038
		***q*_e(theor)_(mg/g)**	0.036	0.202	0.230	0.367	0.266
		***k*_1_ (1/min)**	0.00069	0.00115	0.00046	0.00069	0.00069
		***R***^**2**^	0.986	0.987	0.948	0.971	0.894
**Pseudo-second-order**		***q*_e(theor)_(mg/g)**	0.290	0.529	0.763	0.945	1.071
		***k*_2_ (1/min)**	0.048	0.0109	0.0046	0.0033	0.0049
		***R***^**2**^	0.999	0.999	0.995	0.992	0.999

**Table 2:** Kinetic Parameters for MR Removal by *J. effusus*

			**Initial Dye Concentrations** (mg/L)				
Kinetic Model	Equations (MR)	Kinetic Parameter	10	20	30	40	50
**Pseudo-first-order**		***q*_e(exp)_(mg/g)**	0.270	0.660	0.884	1.155	1.421
		***q*_e(theor)_(mg/g)**	0.138	0.559	0.623	0.881	1.159
		***k*_1_ (1/min)**	0.00069	0.00092	0.00069	0.00092	0.00069
		***R***^**2**^	0.955	0.977	0.960	0.941	0.967
**Pseudo-second-order**		***q*_*e*(theor)_(mg/g)**	0.292	0.748	1.015	1.379	1.746
		***k*_2_ (1/min)**	0.0077	0.0023	0.0012	0.00079	0.00054
		***R***^**2**^	0.998	0.999	0.995	0.994	0.986

### 3.5 Adsorption Isotherms

Adsorption isotherms are graphs that depict the equilibrium between the amount of substance adsorbed onto an adsorbent and the concentration of the substance remaining in the solution at a constant temperature and pH. Langmuir and Freundlich adsorption isotherms are the most commonly used methods to characterize the surface properties of an adsorbent and its interaction with an adsorbed substance. The Langmuir isotherm model assumes that adsorption occurs as a monolayer on a homogeneous surface with all active sites having the same energy and equal affinity for the molecules being adsorbed. In contrast, the Freundlich isotherm model assumes that adsorption is multilayered and that the adsorbent surface is heterogeneous in terms of adsorption sites and energy.^14^ The Langmuir and Freundlich isotherm equations are shown in Table 3. From the Langmuir equation, it is apparent that for a system that obeys this model, plotting *C*_e_/*q*_e_ against *C*_e_ will yield a straight line, while for a system that conforms to the Freundlich equation, a straight line would be obtained from plotting ln *q*_e_ against ln *C*_e_. Accordingly, to assess the nature of the dye adsorption, Langmuir and Freundlich isotherms were generated. The results revealed that the Freundlich isotherms for the MB and MR dyes (Figures S3B and S4B) are a more suitable model to explain dye adsorption by *J. effusus*, as they exhibited higher correlation coefficients (listed in Table 3) compared to the Langmuir isotherms (Figures S3A and S4A). Notably, the 1/*n* value in the Freundlich isotherm describes surface heterogeneity, with the values below 1 in our study indicating that the adsorption surface is heterogeneous.^5,51^

**Table 3:** Isotherm Parameters of MB and MR Removal *by J. effusus*^a^

**Isotherm Model**	**Equations**	**Parameters**	**MB**	**MR**
Langmuir		*q*_max_ (mg/g)	2.618	1.483
		*K*_L_ (L/mg)	2.651	1.05
		*R*_L_	0.707	0.836
		*R*^2^	0.959	0.989
Freundlich		1/*n*	0.8	0.21
		*K*_f_ ((mg/g)(L/mg)^1/n^)	0.152	0.808
		*R*^2^	0.991	0.993

a *q*_max_: maximum adsorption capacity (mg g^–1^); *R*_L_: the separation factor constant; *K*_L_: Langmuir constant; C_0_: (mmol L^-1^) is the initial concentration organic dyes; 1/*n* and *K*_f_ : Empirical constants

### 3.6 Selectivity

**Figure 4:**
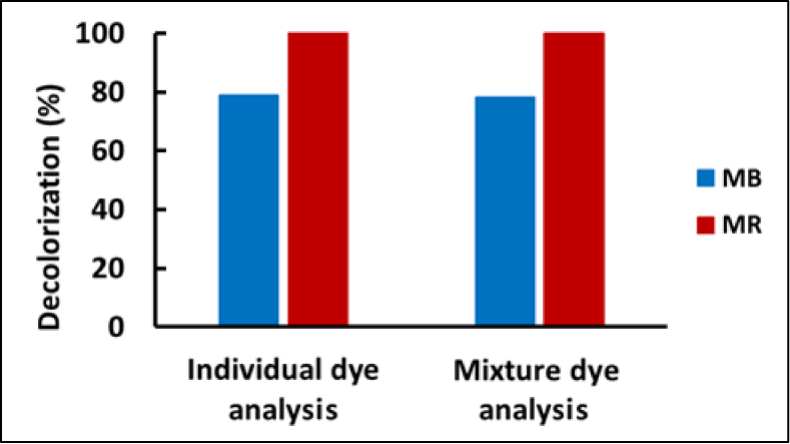
Decolorization percentages observed for *J. effusus*-mediated phytoremediation of water containing MB or MR as single components (i.e., individual dye analysis); or MB and MR as a mixture (mixture dye analysis), at pH 7 using live plants each with a wet weight of ∼1.5 g.

To determine the selectivity of adsorption by *J. effusus* of MB and MR dyes, live plants, each with a wet weight of ∼1.5 g, were exposed for 80 h to 25 mL of a dye mixture containing MB and MR, each at a concentration of 20 mg L^–1^. Absorbance measurements were made at the wavelengths of maximum absorbance for each dye (i.e., 664 for MB and 435 for MR) to determine their concentrations remaining in the water at the end of the monitoring period. Figure 4 shows the results, where the bars on the left show the decolorization percentages obtained when MB (blue) and MR (red) dyes were adsorbed from a solution containing only an individual dye, and the bars on the right show the decolorization percentages for a solution containing equal amounts of the dyes. The results revealed that dye decolorization from the mixture (78.8% for MB and 99.97% for MR) was similar to that observed for the single dyes (78.1% for MB and 99.93% for MR). Thus, in both cases, while all of the MR dye was adsorbed, approximately 80% of the MB dye was removed by *J. effusus*.

### 3.7 Plant Reuse Potential Following Dye Uptake

**Figure 5:**
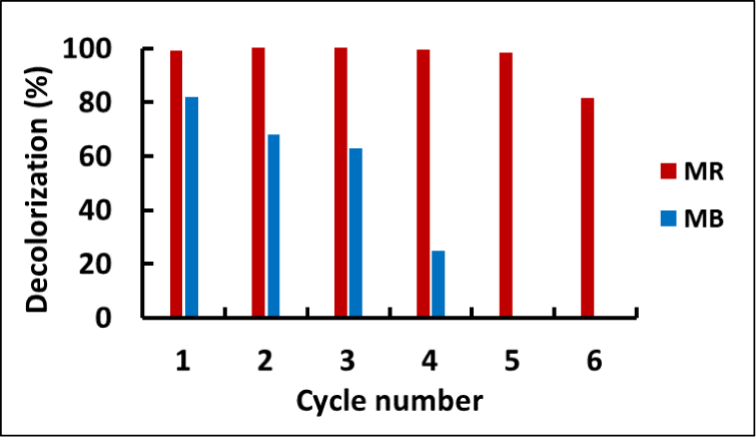
Assessment of the ability of *J. effusus* to adsorb MB and MR dyes over multiple cycles. Dye solutions to which a 1.5 g wet weight plant was exposed were comprised of 50 mL of dye at a concentration of 20 mg L^–1^, pH 7. During each cycle, exposure was for 80 h, and between cycles, plants were kept in Hoagland solution for 24 h before re-exposure of the plant to another dye solution.

To determine the phytoremediation potential of *J. effusus* plants that had previously been used to adsorb MB and MR, their dye uptake was examined over multiple cycles using plants with an approximate wet weight of 1.5 g. Each cycle duration was kept constant at 80 h, and 50 mL solutions of the dyes at a concentration of 20 mg L^–1^ were used. After each cycle, the percentage of decolorization was determined using eq 1, and the plants were kept in Hoagland solution for 1 day before the next cycle. The results are illustrated in Figure 5, with the blue bars representing MB and the red bars representing MR. For MB removal, *J. effusus* exhibited steady decreases in efficiency over the first three cycles, followed by a significant drop in the decolorization percentage in the fourth cycle. For example, in the first, second, and third cycles, the percentage decreases in uptake of MB were 81.9%, 68.1%, and 63.1%, respectively, while in the fourth cycle, it was 24.8%. In the MR removal study, no decrease in decolorization percentage was observed during the first five cycles, indicating that *J. effusus* successfully managed the stress of dye adsorption for five successive cycles. A decrease in the *J. effusus* dye removal capacity to 81.5% was observed in the sixth cycle.

### 3.8 ATR-FTIR Analysis

**Figure 6:**
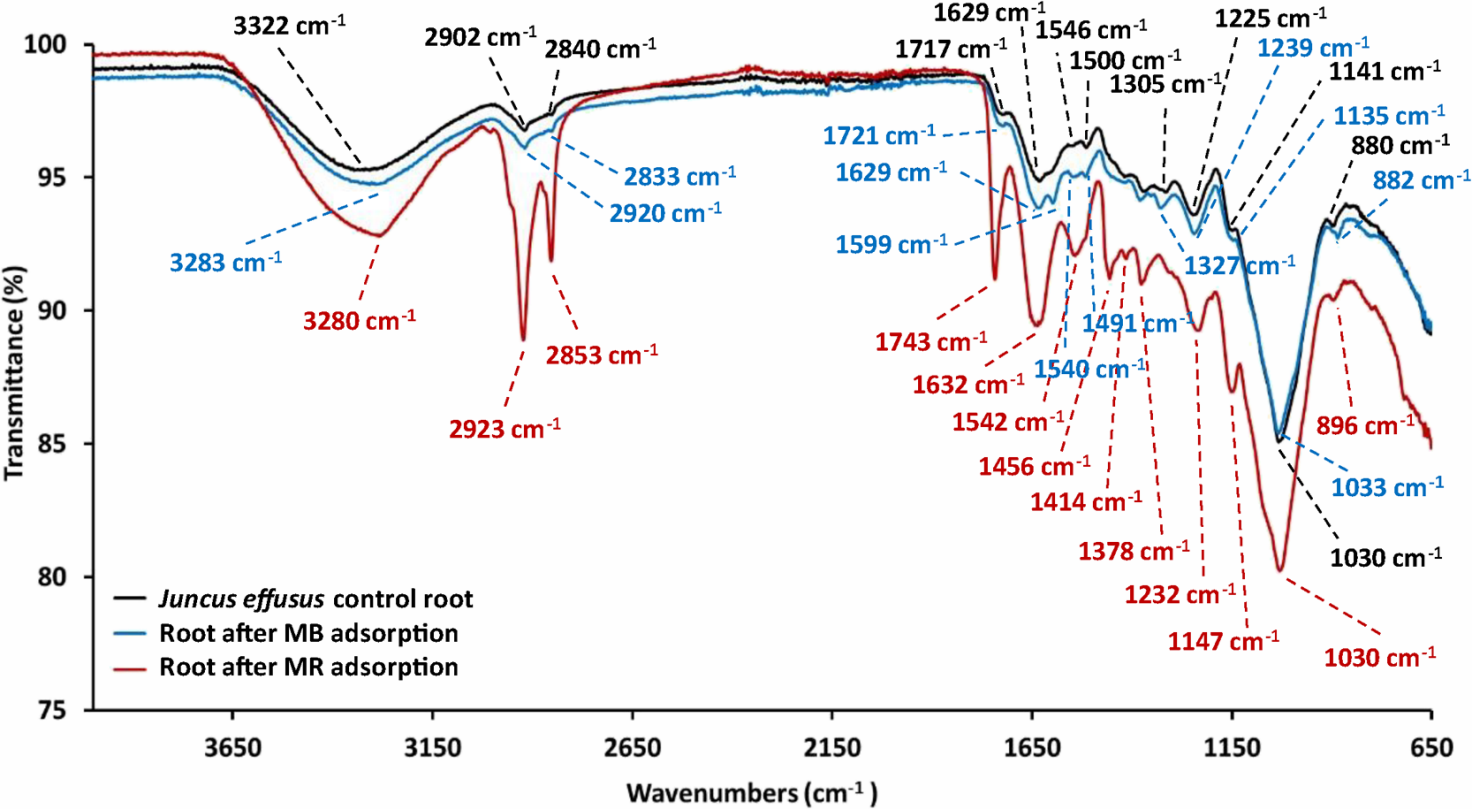
FTIR spectra of *J. effusus* root tissue before (black) and after adsorption of MB and MR dyes (blue and red respectively), highlighting prominent peaks.

**Figure 7:**
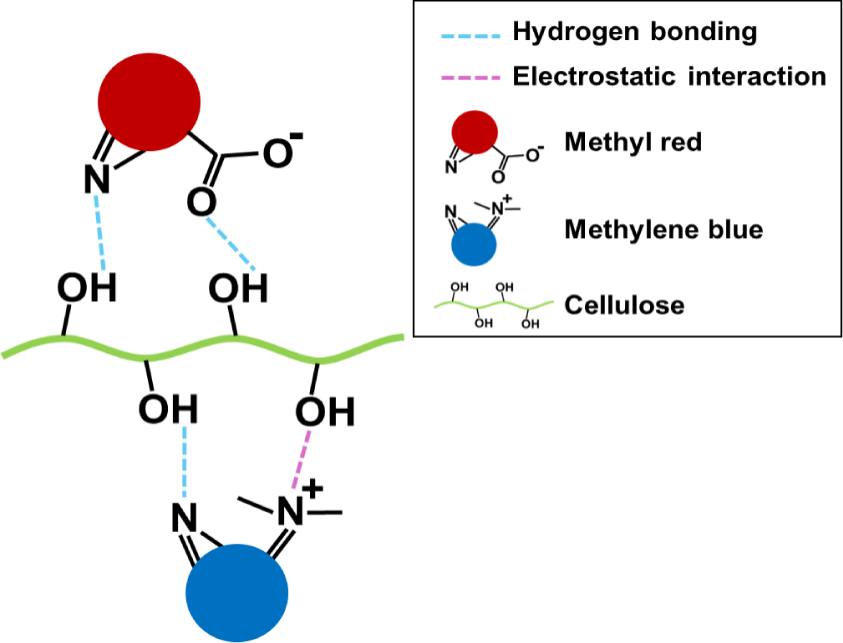
Proposed mechanism for the interaction of MR and MB dyes on *Juncus effusus* roots.

To examine the functional group interactions between MB and MR molecules and the *J. effusus* surface, and provide insight into the phytoabsorption mechanism, FTIR was used. Figure 6 shows the FTIR spectra of *J. effusus* roots before and after adsorption of MB and MR (blue and red colors, respectively). The presence of C–O, C–H, and O–H functional groups is revealed by the absorption bands at 1225, 2902, and 3322 cm^–1^, respectively,^44^ for the control root (black spectrum) that had not been exposed to any dye. These functional groups, which are features of the structures of cellulose, polysaccharides, and carboxylic acids, play a crucial role in the mechanism of removal of organic dyes by plants.^44^Figure 6 indicates that phytoabsorption of the organic dyes results in an intensification of some of the peaks. For example, after phytoabsorption of MB and MR, the peak at 3322 cm^–1^ shifts to 3283 and 3280 cm^–1^, respectively, and becomes broader, owing to intermolecular H-bonding interactions. Additionally, the −CH_2_ stretching band at 2902 cm^–1^ shifts to 2920 cm^–1^ after MB adsorption and to 2923 cm^–1^ following MR adsorption (Table 4). Overall, these observations indicate hydrogen bonding, van der Waals forces, and other interactions between molecules in *J. effusus* roots and those of MB and MR, and the chemical reactions involved in the phytoabsorption process. Other peaks associated with dye adsorption include those between 1629 and 1033 cm^–1^ representing the aromatic ring structure, which shift after dye adsorption;^5^ and those appearing at 1414 and 1456 cm^–1^ (after MR phytoremediation), and 1620 cm^–1^ (after MB phytoremediation). Overall, the results suggest that dye molecules are adsorbed through intermolecular hydrogen bonding as well as electrostatic interactions with dye molecules and functional groups present on the root surface of *J. effusus*. For example, hydrogen bonding interactions may involve the nitrogen of the dye molecule and the hydroxyl groups of cellulose, while electrostatic interactions could occur between the positively charged nitrogen and negatively charged oxygens of cellulose. Therefore, adsorption of MR by *J. effusus* roots occurs primarily through hydrogen bonding, while for MB, adsorption takes place through both hydrogen bonding and electrostatic interactions. Figure 7 illustrates the proposed adsorption mechanisms of MB and MR dyes on *Juncus effusus*.^24,25^ Once adsorbed to the root surface, the dye molecules are then assimilated into the root cells where degradation can occur.

**Table 4:** Functional Group Interactions between *J. effusus* Root Compounds and MB and MR Dyes, as Revealed by FTIR Spectroscopy^a^

**Before Dye Adsorption**	**After MB Adsorption**	**After MR Adsorption**	**Functional Group**	**Reference**
3322	3283	3280	–OH hydroxyl group stretch	(44)
2902	2920	2923	–CH_2_– methylene group stretching	(44)
2840	2833	2853	–CH	(44)
1717	1721	1743	–C=O (nonconjugated stretch)	(24)
1629	1629	1632an	C=C	(44)
	1599		C=O (conjugated stretch)	(24)
1546	1540	1542	N–O stretching	(25)
1500	1491			
		1456	–N =N Stretching	
		1414	C–O stretching	(25)
1305	1327	1378	O–H bending	(24)
1225	1239	1232	C–O carbonyl	(24)
1141	1135	1147	C–O stretch (alcohol)	(24)
1033	1030	1030	C–N stretching	(24)
880	882	896	C–C–H Bending	(25)

a Descriptions of the indicated interactions can be found in the cited references.

### 3.9 DART-HRMS Analysis of Plant Roots Following Dye Removal

**Figure 8:**
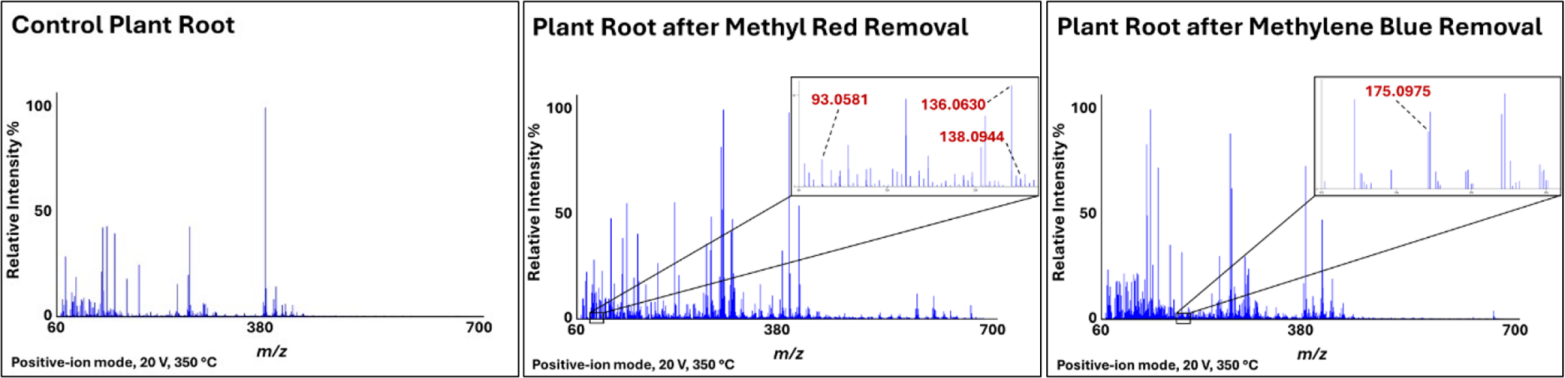
Representative DART mass spectra of *J. effusus* root tissue before and after dye (MR and MB) exposure. High-resolution masses consistent with the presence of aniline, *N*,*N*-dimethyl-*p*-phenylenediamine, and protonated 2-aminobenzoic acid for the MB removal study (middle panel, *m*/*z* 93.0581, 136.0630, and 138.0944, respectively) and that of *m*/*z* 175.0975 (corresponding to 4-(dimethylamino)benzaldehyde) for the MR removal study (last panel) are highlighted.

Patro et al. investigated phytoremediation using macrophytes to remove MR from water. In that study, wastewater samples were collected on different days following exposure to the plants, and the water was analyzed by GC-MS, HPLC, and FTIR. This examination revealed the presence of new molecules introduced into the wastewater, including nitrobenzene, aniline, *N*,*N*-dimethyl-*p*-phenylenediamine, and 2-aminobenzoic acid, which were all proposed to be degradation products of MR metabolism that were released into the water following dye adsorption. Accordingly, an MR degradation mechanism for methyl red based on the observed compounds was proposed.^51^ Similar results were obtained in a study by Jayapal et al.^52^ In our work, after the completion of the dye (MR) removal process using *J. effusus*, the roots were analyzed directly by DART-HRMS to track the presence of masses consistent with those of metabolites detected in the aforementioned earlier studies. The results of the DART-HRMS analysis of the roots, following their uptake of the dyes, are presented in Figure 8. They show the presence of high-resolution masses consistent with formulas associated with: (1) *N*,*N*-dimethyl-*p*-phenylenediamine (C_8_H_12_N_2_; nonprotonated); (2) 2-aminobenzoic acid (C_7_H_7_NO_2_ + H^+^); and (3) aniline (C_6_H_7_N; nonprotonated) (i.e., nominal *m*/*z* 136, 138, and 93, respectively). No such masses were observed in control root tissue that was not exposed to the dyes (Figure 8). These findings suggest that degradation of MR occurred in the roots of the plants.

With regard to MB, Imron et al. observed that phytoremediation from contaminated water using *Lemna minor* resulted in metabolism that furnished 4-(dimethylamino)benzaldehyde as a byproduct, and the authors proposed a degradation mechanism involving a desulfurization process for MB.^44^ In our study, DART-HRMS analysis of *J. effusus* root tissue before and after exposure to MB-contaminated water revealed a high-resolution mass consistent with the presence of 4-(dimethylamino)benzaldehyde (C_11_H_13_NO; nonprotonated, nominal mass at *m*/*z* 175) in roots exposed to the dye (Figure 8). This mass was not observed in the roots of plants that were not used for phytoremediation. These findings lend support to the pathway proposed by Imron et al. for MB degradation and may indicate that plants of various species metabolize MB via a common mechanism. The DART mass spectral data tables corresponding to the spectra in Figure 8 are listed in Tables S1, S2, and S3.

### 3.10 Comparison of the Dye Removal Potential of Vascular Plants

A number of studies have been conducted to explore the utility of live plants to remove organic dyes from contaminated water, and it has been shown that a variety of plants exhibit this capacity (Table 5). Generally, such studies are conducted within pH ranges suitable for plant viability (slightly acidic to neutral), and neutral pH is advantageous for working with natural water sources. Previous studies have shown that the plants that remove dyes from water conform to the Freundlich isotherm, which indicates that the adsorption surface is heterogeneous. *Chara vulgaris*, *Lemna minor* and *Trachyspermum ammi* have been studied for the removal of MR and MB. *C. vulgaris* is a freshwater green alga species; *L. minor* is a floating freshwater aquatic plant; and *T. ammi* is an annual terrestrial plant. At their pH’s of optimal adsorption, these plants take up 0.899 mg g^–1^ (for MR), 1.14 mg g^–1^ (for MB), and 0.135 mg g^–1^ (for MB), respectively (see Table 5).^24,25,44^ We observed the adsorption values for *J. effusus* to be higher in comparison (see Table 5). Moreover, we show that *J. effusus* is effective in the phytoremediation of both dyes.

**Table 5:** Comparison of Different Phytoremediation Studies for Wastewater Dye Removal

**Dye**	**Plant species**	**pH**	**Isotherm**	*q***_max_** (mg/g)	**Duration time (h)**	**Reference**
MR	*Chara vulgaris*	5	Freundlich	0.899	48	(25)
MB	*Lemna minor*	6−7.5	Freundlich	1.14	96	(44)
Congo Red	*Bryophyllum fedtschenkoi*	5	Freundlich	0.925	40	(5)
Congo Red	*Trachyspermum ammi*	5	Freundlich	0.150	40	(24)
MB	*Trachyspermum ammi*	8	Freundlich	0.135	40	(24)
Crystal violet	*Salvinia molesta*	6		1.073	120	(23)
Direct Red 28	*Salvinia molesta*	6.5		1.087	120	(48)
MB	*Juncus effusus*	7.5	Freundlich	1.038	80	This work
MR	*Juncus effusus*	7	Freundlich	1.421	80	This work

### 3.11 Visual Detection of Adsorption of Dyes by Live *J. effusus* Plants

**Figure 9:**
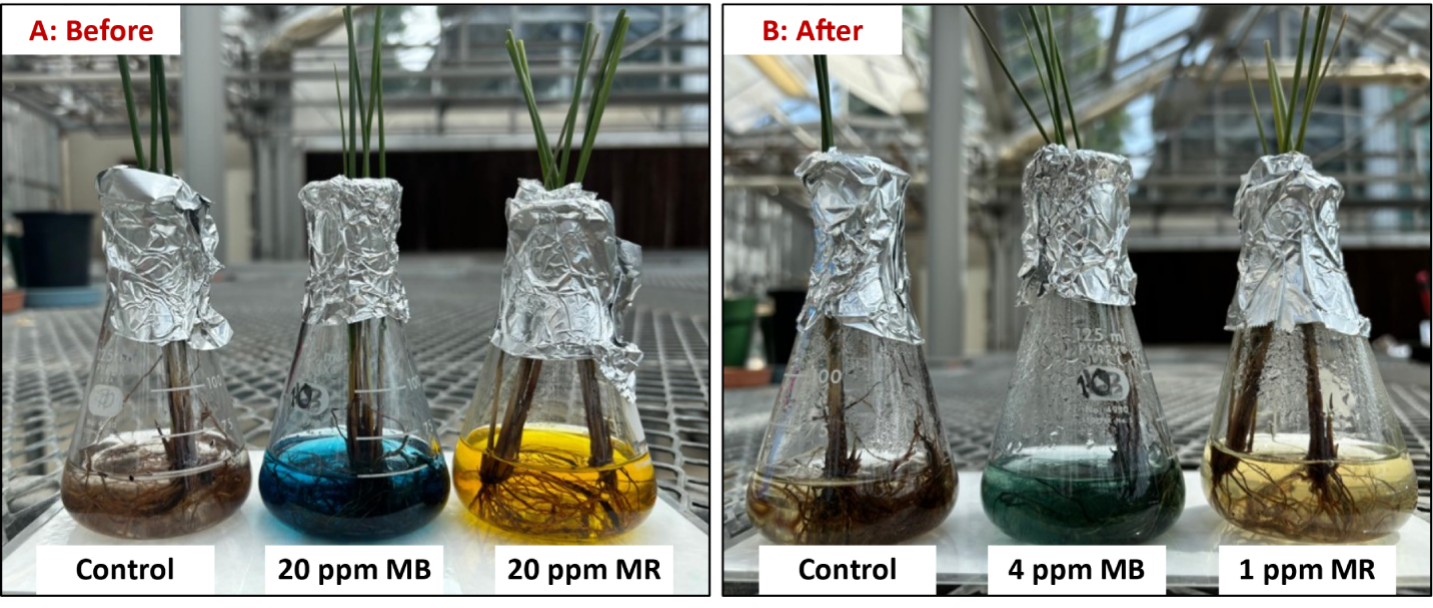
Visual detection of decolorization of dye-contaminated water by *J. effusus* roots. A: control roots (no added dye), 20 ppm of MB and 20 ppm of MR; B: control roots (no dye added) and MB and MR solutions following exposure to *J. effusus* roots after 80 h at pH 7. Each plant has a biomass of ∼1.5 g of wet weight.

As the dyes are colored, their uptake by the plants could be perceived visually by the decrease in the intensity of the color of the dye solutions to which the plants were exposed. This is illustrated in Figure 9. In Figure 9A, the water into which *J. effusus* roots were suspended at the start of the exposure contained 20 ppm of MR and appeared orange in color (left-hand side). However, after 80 h, the solution is significantly lighter in color (Figure 9B). A similar trend was observed with MB. When *J. effusus* was first exposed to the MB-spiked water at a concentration of 20 ppm, the solution was dark blue in color (Figure 9A). However, after 80 h, the intensity of the blue color was substantially reduced (Figure 9B). At the same time, the roots of the plants that were exposed to the dye solutions had taken on the color of the respective dyes (image not shown).

## 4 Conclusions

Phytoremediation studies were conducted using the plant *J. effusus* to treat methylene blue and methyl red contaminated water. The impact of various factors such as pH, plant biomass, contact time, and initial dye concentration was investigated. While the maximum decolorization percentages were observed at a dye concentration of 10 ppm (i.e., ∼79% for MB and ∼100% for MR), the maximum amount of dye removed from the solution (i.e., the amount of dye taken up by the plant) was observed at a concentration of 50 ppm (1.421 and 1.038 mg g^–1^ plant wet weight for MB and MR, respectively). It was found that the Freundlich model, with a correlation coefficient of *R*^2^ ≥ 0.99, was suitable for describing the adsorption mechanism of MB and MR on the surface of *J. effusus* and that a pseudo-second-order kinetic model best explained the phytoremediation process of MB and MR dyes. The FTIR results showed that MB and MR dyes were first adsorbed to the surface of *J. effusus* through electrostatic interactions and hydrogen bonding, and this was followed by transfer into the cells of *J. effusus*, where they were further degraded. Degradation of the adsorbed dyes was confirmed by DART-HRMS analysis. A mass consistent with 4-(dimethylamino)benzaldehyde, a known byproduct of degraded MB, was observed in the mass spectrum obtained by the analysis of *J. effusus* roots after exposure to an MB-containing solution. The mass spectrum of *J. effusus* roots after exposure to an MR-containing solution revealed masses consistent with *N*,*N*-dimethyl-*p*-phenylenediamine, 2-aminobenzoic acid, and aniline, all previously demonstrated to be metabolites of MR. The results show *J. effusus* to be an effective plant for phytoremediation. The plants’ high decolorization capacity indicates its potential use in water purification for the adsorption of MB and MR, and possibly other organic dyes. Cultivation of *J. effusus* in polluted streams containing dye wastewater may offer a sustainable solution for wastewater treatment.

Overall, this study demonstrates the potential of *J. effusus* for real-world water treatment applications. By utilizing a natural, sustainable method for removing hazardous dyes from aqueous environments, this approach can be readily scaled to suit larger water systems. The ecological impact is also promising, as *J. effusus* is a resilient plant that thrives under a range of environmental and geographic conditions, making it ideal for integration into constructed wetlands or natural filtration systems. Its adoption for this purpose would help to reduce reliance on current chemical treatments that may harm aquatic ecosystems.

## Supporting Information Available

Kinetic study, adsorption isotherm graphs, DART MS, and DART high-resolution mass data table (PDF)
