# Supplementary material for: Efficient Phytoremediation of Methyl Red and Methylene Blue Dyes from Aqueous Solutions by Juncus effusus
Source: ACS Omega. 2025 Jan 10;10(2):1943–53. doi: 10.1021/acsomega.4c07468 (PMC11755181; doi:10.1021/acsomega.4c07468)
Supplement: Supplementary file 1 — ao4c07468_si_001.pdf [file ao4c07468_si_001.pdf]

Supporting Information:

Efficient Phytoremediation of Methyl Red and Methylene Blue Dyes from Aqueous Solutions by *Juncus effusus*

AUTHORS: Oya Aydın Urucu[1,2], Benedetta Garosi[1,i] and Rabi A. Musah[1,i]*

[1]Department of Chemistry, University at Albany – State University of New York (SUNY), 1400 Washington Avenue, Albany, NY 12222, USA

[2]Marmara University Faculty of Sciences, Department of Chemistry, Istanbul 34722, Turkey

*Corresponding author: rmusah@albany.edu (R. A. Musah)

The Supporting Information includes (8 Pages total):

Figure S1. Pseudo-first- and second-order kinetic model for MB dye removal.

Figure S2. Pseudo-first- and second-order kinetic model for MR dye removal.

Figure S3. Langmuir isotherm and Freundlich isotherm for MB dye removal.

Figure S4. Langmuir isotherm and Freundlich isotherm for MR dye removal.

Table S1. DART–high-resolution mass data table for control plant roots.

Table S2. DART–high-resolution mass data table for the plant roots analyzed after methyl red adsorption.

Table S3. DART–high-resolution mass data table for the plant roots analyzed after methylene blue adsorption.

[i] Current address: Department of Chemistry, Louisiana State University, Baton Rouge, Louisiana 70803, USA

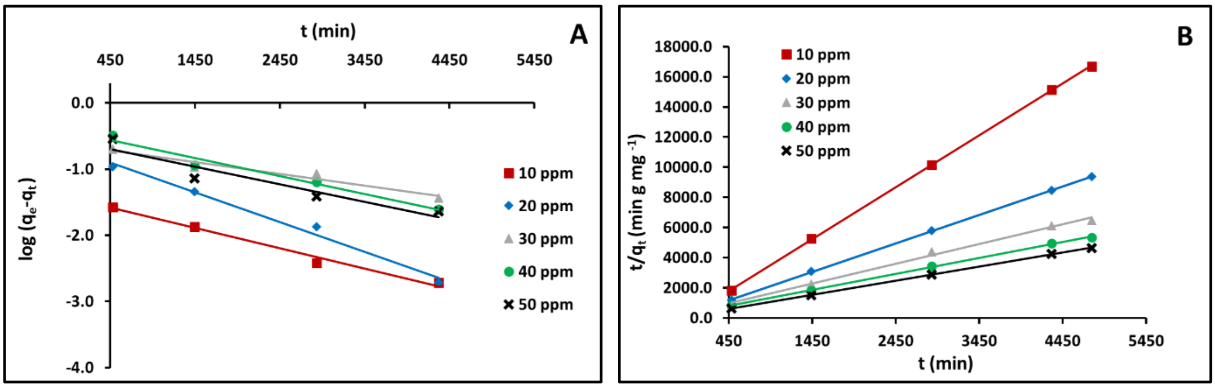

Figure S1. Panel A: Pseudo-first-order kinetic model for MB dye phytoremediation by *Juncus effusus*; Panel B: Pseudo-second-order kinetic model for MB dye phytoremediation by *Juncus effusus*.

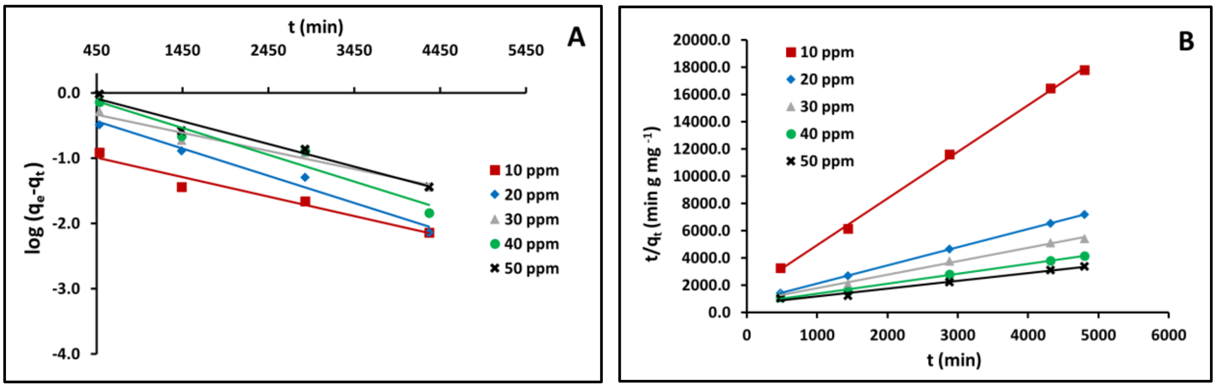

Figure S2. Panel A: Pseudo-first-order kinetic model for MR dye phytoremediation by *Juncus effusus*; Panel B: Pseudo-second-order kinetic model for MR dye phytoremediation by *Juncus effusus*.

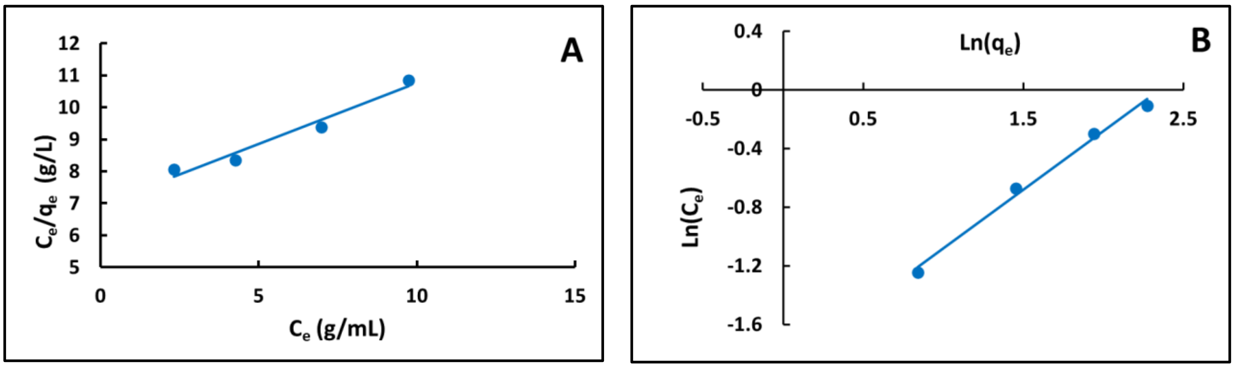

Figure S3. Panel A: Langmuir isotherm for MB dye removal (R^2 = 0.959); Panel B: Freundlich isotherm for MB dye removal (R^2 = 0.991).

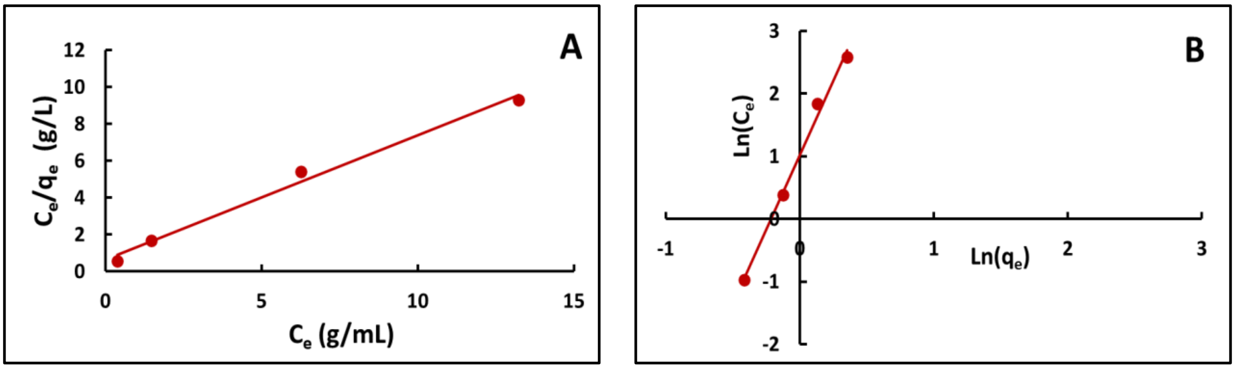

Figure S4. Panel A: Langmuir isotherm for MR dye removal (R^2 = 0.989); Panel B: Freundlich isotherm for MR dye removal (R^2 = 0.993).

Table S1. Mass data table for representative control plant roots. Peak abundances above a 2.0% relative abundance threshold are listed.

m/z	**Rel. Int. (%)**	*m/z*	**Rel. Int. (%)**	*m/z*	**Rel. Int. (%)**
61.0295	3.18	137.1312	2.91	397.3781	5.68
69.0333	3.40	139.1136	2.42	402.3155	6.09
69.0698	8.41	141.0916	3.84	403.3257	2.31
70.0679	2.63	141.1288	2.72	411.3668	2.33
71.0504	4.83	143.1074	3.36	413.3840	5.52
71.0833	5.68	145.1153	3.27	419.3208	2.25
73.0649	28.74	147.0653	39.77		
75.0438	7.90	148.0711	2.11		
81.0703	3.56	149.1202	2.25		
83.0850	11.58	155.1029	2.93		
84.0446	7.03	155.1447	2.04		
84.0799	2.35	157.1269	2.01		
85.0290	3.89	165.0769	18.19		
85.0644	7.33	183.0858	24.87		
87.0433	9.88	201.1772	2.09		
89.0624	19.01	229.2169	2.96		
91.0517	5.24	239.2410	2.62		
93.0671	2.46	241.1812	15.51		
95.0845	5.37	255.2293	3.53		
97.0636	2.18	257.2472	19.96		
97.0981	8.16	258.2480	2.66		
99.0464	4.72	259.1894	42.88		
99.0771	7.52	260.1975	5.65		
101.0603	9.08	263.2443	2.95		
103.0399	2.84	271.2407	2.04		
109.0997	8.41	275.2664	2.42		
111.1184	7.27	279.2325	6.62		
113.0640	2.44	281.2446	5.94		
113.0954	4.26	283.2644	6.07		
115.0806	4.42	285.2786	4.15		
117.0934	6.79	295.2257	2.03		
123.1158	3.59	298.2793	2.37		
125.0967	6.28	369.3093	7.02		
125.1267	2.53	371.5768	5.08		
127.1107	21.46	371.6746	2.73		
129.0526	42.59	372.3166	100		
129.1243	4.36	373.3213	13.41		
130.0535	12.40	374.3240	2.14		
130.0833	9.47	385.3019	8.49		
131.0983	2.94	387.3211	3.83		
135.1024	43.26	388.3420	14.34		
136.1108	2.03	389.3510	2.68		

**Table S2.** Mass data table for representative plant roots that were analyzed after methyl red removal. Peak abundances above a 2.0% relative abundance threshold are listed.							
m/z	**Rel. Int. (%)**	***m/z***	**Rel. Int. (%)**	***m/z***	**Rel. Int. (%)**	***m/z***	**Rel. Int. (%)**
61.0311	3.70	115.0394	6.45	157.1263	6.10	241.1681	5.10
69.0334	9.54	115.0812	8.65	165.0801	16.48	243.2100	2.41
69.0697	8.28	116.0708	4.27	167.1092	6.05	245.1426	3.62
70.0673	9.98	117.0562	16.93	170.1081	2.45	245.2242	4.03
71.0505	4.79	117.0904	10.23	173.1218	7.95	247.2359	3.25
71.0832	6.38	118.0876	2.35	175.1517	2.12	251.1794	2.94
73.0665	18.08	121.0600	2.65	179.0768	8.10	253.0870	12.53
75.0455	22.54	121.0985	3.23	179.1436	2.83	253.2070	3.59
81.0334	4.69	123.0599	2.71	180.0982	3.95	255.2325	11.66
81.0701	5.37	123.1156	6.99	181.1172	2.56	256.2404	2.15
83.0866	12.51	125.0998	6.18	183.0886	26.65	257.2471	35.90
84.0446	16.51	125.1326	3.94	184.0925	2.06	258.2511	5.30
84.0795	9.64	126.0598	3.35	185.1273	2.72	259.1931	11.12
85.0291	5.75	126.0930	3.29	187.1385	2.02	261.2144	2.31
85.0645	7.72	127.0477	5.33	189.1204	4.11	263.2396	32.82
86.0602	28.11	127.1100	7.88	189.1597	2.82	264.2430	7.76
87.0453	12.14	129.0573	21.54	190.1497	4.92	265.2527	48.69
89.0625	12.66	130.0526	38.70	191.1840	2.18	266.2581	8.85
90.0570	7.63	131.0722	2.95	192.0799	8.82	267.2654	3.42
90.0870	3.11	132.1007	3.32	193.0873	3.03	268.1091	6.65
91.0534	3.94	133.0880	2.95	193.1439	3.83	269.0976	2.74
93.0581	14.94	136.0630	55.25	195.1277	6.40	269.2104	3.95
95.0863	9.05	136.1038	8.61	197.1583	2.40	271.2346	4.16
96.0835	2.03	137.1185	6.02	199.1716	2.54	274.2765	2.23
97.0305	5.74	138.0598	4.25	201.1451	2.05	275.2620	4.33
97.0652	2.65	138.0944	3.30	207.1336	2.79	277.2164	20.14
97.0981	8.58	139.1135	6.98	209.0816	55.68	278.2244	4.77
99.0443	22.89	140.0783	2.01	210.0883	7.28	279.2316	82.21
99.0765	9.61	141.0979	3.71	210.1529	2.25	280.2387	18.39
100.0772	2.02	143.1098	6.02	211.1395	6.49	281.2452	52.15
101.0604	6.78	143.1414	2.31	216.0896	20.95	282.2509	9.66
103.0398	3.44	145.0550	3.45	217.1998	2.12	283.2610	100
103.0852	9.51	145.1128	2.44	225.1704	2.76	284.2658	19.87
104.0708	10.23	147.0680	19.63	227.1002	6.93	285.2777	10.99
106.0526	3.41	148.0748	2.52	229.1489	7.09	286.2667	2.40
109.1017	9.67	150.1148	3.42	229.2067	4.16	291.2011	8.74
110.0804	8.26	151.1129	3.79	231.1644	3.20	293.2129	16.18
111.1184	6.79	151.1475	4.03	232.0848	4.66	294.2218	3.74
112.0499	48.02	153.0778	40.68	233.1697	2.11	295.2289	41.66
112.0859	28.07	154.0896	3.22	237.2251	7.07	296.2364	9.36
113.0532	5.70	155.1055	18.19	239.1696	2.24	297.2439	47.57
113.0950	5.57	156.0825	4.27	239.2399	7.33	298.2693	21.86

**Table S2 (continued).** Mass data table for representative plant roots analyzed after methyl red removal. Peak abundances above a 2.0% relative abundance threshold are listed.			
m/z	**Rel. Int. (%)**	***m/z***	**Rel. Int. (%)**
299.2687	13.04	396.3448	2.17
300.2890	14.77	397.2370	2.73
301.2939	3.38	397.3718	5.97
309.2138	4.71	398.2331	53.97
311.2343	9.60	399.2375	16.38
312.2482	3.00	400.2509	2.43
313.2733	12.66	409.3555	4.72
314.2817	3.28	411.3604	5.77
327.2209	3.94	412.3778	2.36
329.2616	2.18	413.3799	4.84
330.2708	2.63	415.2629	2.09
331.2904	7.25	425.3753	3.62
332.2887	2.75	427.3642	3.42
337.2852	4.64	429.3467	3.56
339.2899	16.50	467.3808	2.67
340.2990	4.02	493.3967	2.06
341.3020	3.92	519.4826	2.07
351.2661	2.87	521.4977	3.15
353.2745	6.83	538.5317	2.16
354.2812	2.17	551.5196	2.47
355.2847	5.11	575.5146	4.86
356.2989	2.21	577.5257	12.02
357.3014	11.08	578.5271	4.96
358.3123	2.68	579.5259	2.19
359.3203	2.29	601.5320	5.08
367.2937	2.31	602.5390	2.22
369.0719	4.82	603.5443	10.84
369.3517	9.42	604.5445	4.42
370.3495	2.58	605.5574	4.08
371.5810	2.15	617.5257	3.45
372.3202	32.51	619.5425	2.34
373.3209	5.37	628.3831	4.06
375.3061	4.05	659.4136	6.56
377.3266	5.14	660.4279	2.72
383.0738	98.64		
383.3524	2.94		
384.0808	21.67		
385.0954	5.01		
385.2760	2.61		
388.3473	2.85		
393.3236	3.23		
395.3438	5.61		

Table S3. Mass data table for representative plant roots analyzed after methylene blue removal. Peak abundances above a 2.0% relative abundance threshold are listed.

m/z	Rel. Int. (%)	*m/z*	Rel. Int. (%)	*m/z*	Rel. Int. (%)	*m/z*	Rel. Int. (%)
67.0544	3.07	103.0400	5.06	132.1007	7.24	169.1350	5.38
69.0334	16.32	103.0768	4.76	133.0917	8.59	171.1465	9.28
69.0695	10.74	104.0708	9.07	135.1026	100	173.1231	3.27
70.0670	23.66	105.0703	2.38	135.2452	4.95	175.0975	2.10
71.0505	4.02	106.0501	17.86	136.0656	10.11	175.1488	2.82
71.0833	6.81	107.0729	4.06	136.1079	8.71	177.0577	2.73
72.0809	11.17	108.0818	2.15	137.1334	9.09	177.1419	3.48
73.0649	15.55	109.0999	21.31	138.0603	2.88	179.0789	3.00
74.0607	6.60	110.0677	2.07	138.0916	2.55	179.1441	2.87
74.0943	3.82	110.1008	3.53	139.0515	25.85	179.1792	2.50
75.0440	4.04	111.0434	18.28	139.1137	8.99	181.1066	2.02
76.0402	3.27	111.0789	4.28	140.0718	3.06	181.1584	2.64
81.0719	10.01	111.1184	13.85	141.0978	5.03	182.0893	2.35
83.0498	4.65	112.0498	19.39	141.1608	2.45	183.0884	32.01
83.0866	17.73	112.1176	3.99	142.0845	2.06	184.0923	2.93
84.0447	18.42	113.0316	3.31	143.1075	6.12	185.1359	3.13
84.0795	10.09	113.0640	6.32	144.0907	2.54	187.1447	2.64
85.0291	11.58	113.0953	5.95	145.0528	5.43	189.1213	2.82
85.0623	9.32	113.1302	3.68	145.1100	3.65	189.1640	3.34
85.0992	4.18	114.0616	3.43	146.0883	2.44	191.1706	2.97
86.0603	11.89	114.0928	4.56	147.0652	72.23	192.0846	2.38
86.0934	8.42	115.0396	11.06	148.0707	5.90	193.0905	5.52
87.0434	18.01	115.0838	21.92	149.1215	9.25	193.1443	3.61
88.0769	3.45	116.0710	21.20	150.1150	5.01	193.1893	2.79
89.0623	17.01	117.0933	8.54	151.1049	4.99	195.1205	4.98
90.0571	11.49	118.0877	5.76	151.1478	7.04	197.1590	2.82
90.0847	3.75	119.0874	2.63	152.0633	2.73	199.1717	3.88
90.1064	2.01	120.0697	9.81	152.1282	6.07	201.1771	4.04
91.0516	4.56	121.0447	7.81	153.0615	5.61	203.1611	2.22
93.0672	4.36	121.0984	6.19	153.1289	5.73	205.1988	2.15
95.0845	21.66	123.1158	13.27	155.1029	6.98	207.1255	2.24
96.0859	3.29	125.0969	9.83	155.1445	4.76	209.0872	2.34
97.0305	9.64	125.1297	8.13	156.0791	4.87	209.1512	3.64
97.0631	5.07	126.0571	3.27	157.1263	6.36	211.1609	4.30
97.0983	15.63	126.0933	2.15	159.1027	2.48	216.0934	3.92
98.0975	3.17	127.0452	7.76	161.0987	2.58	217.1873	3.24
99.0464	17.77	127.1085	19.11	161.1340	2.76	219.1369	2.06
99.0767	10.49	128.0755	4.25	163.1293	5.92	219.1962	2.10
100.0726	3.87	128.1117	3.99	165.0768	35.47	221.1588	2.30
101.0604	13.52	129.0548	83.34	166.0859	5.83	221.2213	2.64
101.0869	3.98	130.0506	49.46	166.1711	2.33	223.2081	2.40
102.0874	4.55	131.0850	4.93	167.1037	5.55	225.2022	3.21

**Table S3 (continued).** Mass data table for representative plant roots analyzed after methylene blue removal. Peak abundances above a 2.0% relative abundance threshold are listed					
m/z	**Rel. Int. (%)**	***m/z***	**Rel. Int. (%)**	***m/z***	**Rel. Int. (%)**
226.1816	2.14	281.2450	16.75	388.3476	3.32
227.1889	3.36	282.2512	3.13	393.3377	2.83
229.2192	12.25	283.1805	4.11	395.3653	10.05
231.1652	2.67	283.2642	21.04	396.3686	3.80
231.2129	2.58	283.4023	2.39	397.3813	47.47
232.0849	2.63	284.2716	4.85	398.2369	23.23
233.1238	2.70	285.1370	5.55	398.3861	14.80
233.2071	2.46	285.2782	24.09	399.2425	7.77
235.1994	4.70	286.1377	10.67	399.3583	7.22
237.2256	7.84	286.2720	6.22	400.3546	2.54
239.1556	3.64	287.2081	2.34	401.3525	2.01
239.2405	8.86	291.2079	2.06	402.3299	2.59
241.1812	30.01	293.2224	5.08	403.3246	3.77
242.1825	4.85	295.2260	9.11	409.3603	3.96
243.2302	9.44	296.2463	2.52	411.3600	8.89
244.2181	3.75	297.2526	8.29	412.3666	3.32
245.2247	3.79	298.2784	5.75	413.3748	19.12
247.2356	2.70	299.2737	7.66	414.3863	5.74
251.1928	3.12	300.2934	3.39	415.3412	3.55
253.2184	4.85	303.2922	2.54	425.3813	2.83
255.2289	11.22	311.2379	3.85	427.3629	5.45
256.2526	3.00	313.2789	6.24	429.3595	7.63
257.2471	88.47	327.2159	5.66	430.3787	2.53
258.2513	14.24	329.2710	2.70	431.3681	2.44
259.1894	62.39	331.2928	2.77	445.3716	2.48
260.1975	9.13	338.3506	2.76	659.4211	3.92
261.2218	3.00	341.3018	2.30		
263.2402	15.44	357.3038	2.28		
264.2432	2.74	367.3401	2.97		
265.2560	12.16	369.3520	17.11		
266.2590	2.62	370.3587	4.23		
267.2558	4.46	371.5810	4.36		
269.2300	4.03	372.3163	73.00		
270.1065	4.20	373.3253	9.88		
271.1168	3.78	375.3011	2.87		
271.2411	6.98	377.3234	4.99		
272.1242	4.78	379.3474	3.01		
272.2486	3.15	381.3571	2.01		
275.2653	7.61	383.3652	10.96		
277.2168	9.29	384.3793	3.67		
279.2351	29.82	385.3105	6.52		
280.2405	6.09	387.3267	2.94		
